# Gabapentin versus Transdermal Fentanyl Matrix for the Alleviation of Chronic Neuropathic Pain of Radicular Origin: A Randomized Blind Multicentered Parallel-Group Noninferiority Trial

**DOI:** 10.1155/2019/4905013

**Published:** 2019-02-04

**Authors:** Chang Ju Hwang, Jae Hyup Lee, Jung-Hoon Kim, Sang Hyuk Min, Kun-Woo Park, Hyoung-Yeon Seo, Kwang-Sup Song

**Affiliations:** ^1^Department of Orthopaedic Surgery, Asan Medical Center, University of Ulsan College of Medicine, Seoul, Republic of Korea; ^2^Department of Orthopaedic Surgery, Seoul National University College of Medicine, Seoul, Republic of Korea; ^3^Department of Orthopaedic Surgery, Inje University Ilsan Paik Hospital, Inje University College of Medicine, Goyang, Republic of Korea; ^4^Department of Orthopaedic Surgery, Dankook University Hospital, Cheonan, Republic of Korea; ^5^Department of Orthopaedic Surgery, Bundang Chuk Hospital, Seongnam, Republic of Korea; ^6^Department of Orthopaedic Surgery, Chonnam National University Hospital, Gwangju, Republic of Korea; ^7^Department of Orthopaedic Surgery, Chung-Ang University College of Medicine, Seoul, Republic of Korea

## Abstract

A number of studies have been published proposing various approaches to the treatment of neuropathic pain; however, to our knowledge, no attempts have been made to compare gabapentin and fentanyl in patients with lumbar radiculopathy. We evaluated the relative efficacy and safety of fentanyl matrix and gabapentin for the treatment of chronic neuropathic pain of radicular origin. The study was designed as a randomized blind multicentered parallel-group noninferiority trial. A total of 108 patients with moderate-to-severe pain (≥4 intensity on an 11-point numeric rating scale) were randomly prescribed either fentanyl matrix or gabapentin over a period of 56 days. In the primary analysis, the noninferiority of fentanyl matrix treatment was evaluated in relation to the efficacy of gabapentin based on the pain intensity difference (PID) at 56 days after the first dose of the drugs. Secondary endpoints included pain relief, improvement in functional status (the Korean-Oswestry Disability Index (K-ODI)), improvement in depressive symptoms (Korean-Beck Depression Index (K-BDI)) between the 28th and 56th day, and adverse events (AEs). Analysis of the primary efficacy endpoint established the noninferiority of fentanyl matrix compared with gabapentin, with no statistically significant difference observed in the PID after 56 days for the two treatment groups. Similarly, analysis of pain relief revealed no significant differences between the groups on days 28 and 56. There was no difference in the K-ODI and K-BDI between the groups during the study period. The overall incidence of at least one AE was similar for fentanyl matrix (67.3%) and gabapentin (69.6%). The most commonly reported AEs for patients treated with fentanyl matrix and gabapentin included dizziness (30.8% vs. 44.6%, respectively), somnolence (26.9% vs. 35.7%), and constipation (15.4% vs. 17.9%). This study demonstrated that the analgesic effect of fentanyl matrix is noninferior in comparison with gabapentin and supports the use of fentanyl matrix as an effective and safe treatment for moderate-to-severe chronic neuropathic pain. This trial is registered with NCT01127100.

## 1. Introduction

Neuropathic pain is defined as “pain caused by a lesion or disease of the somatosensory system” [1]. It has various causes, including lumbar radiculopathy, which is often responsible for low back pain with sciatica, one of the most commonly encountered symptoms in primary care settings.

Because of its ubiquity, various medications are used to treat neuropathic pain. Gabapentin is an anticonvulsant that is recommended most commonly for neuropathic pain due to its efficacy for diabetic neuropathy, postherpetic neuralgia, and other neuropathic conditions as demonstrated in several randomized controlled trials (RCTs) [2–4].

Opioids, which are the most effective broad-spectrum analgesic for acute pain, have increasingly been used to treat neuropathic pain, even though controversy remains over their efficacy and safety [5–23]. An example of this is fentanyl, which is a significantly more potent opioid than morphine that was first synthesized in the 1950s [18]. It was used initially for intravenous anesthesia and analgesia, but its prescription for chronic pain is increasing with the recent development of a transdermal formulation. Fentanyl patch is safer for patients with renal insufficiency, an effective substitute for patients who cannot tolerate fentanyl in its oral formulation, and less likely to cause constipation compared to conventional opioids.

Several studies have reported on the efficacy of medications for the treatment of neuropathic pain, but to our knowledge, no attempts have been made to compare gabapentin and fentanyl in the treatment of pain arising from lumbar radiculopathy. With a focus on the safety and convenience of transdermal fentanyl, we planned an RCT comparing gabapentin and transdermal fentanyl for chronic neuropathic pain in a noncancer setting.

## 2. Materials and Methods

This clinical trial was conducted using a single protocol at seven hospitals, each with its own clinical investigator from May 26, 2010, to November 28, 2011. Based on the inclusion/exclusion criteria described below, 108 patients were selected and stratified by site and randomized into the study drug or comparator group at a 1 : 1 ratio using stratified block randomization. Subject questionnaires were assessed by a rater who was blind to the randomization process.

Patients were enrolled if they complained of chronic neuropathic pain for more than three months before the study began and if they were at least 20 years old. Patients were considered for inclusion only if their mean pain intensity for the three days before entering the study entry was higher than 4 on a numeric rating scale (NRS) and if they were judged by the investigator to be able to follow the overall study process and to complete the questionnaires.

The criteria for the diagnosis of neuropathic pain in the subjects were pain radiating into the corresponding dermatome, motor or sensory change, a symptomatic area that was consistent with an anatomical lesion as observed in magnetic resonance imaging, radiculopathy that was confirmed with electromyography (EMG) test, and a score of more than 12 on the self-reported version of the Leeds Assessment of Neuropathic Symptoms and Signs (S-LANSS).

Patients were excluded if they had received treatment with gabapentin, pregabalin, fentanyl matrix, or long-acting oral opioid analgesics within the 30 days before the trial began; had a history or a laboratory discovery of neuropathy of other origins (e.g., diabetes, herpes zoster, hyperthyroidism, vitamin B12 deficiency, connective tissue disease); had a concurrent disease causing other pain that was more intense than the neuropathic pain; and if other opioid analgesics were contraindicated. Patients who were not expected to provide written consent, who could not understand the warnings, cautions, or contraindications in the instructions, and whose welfare might be threatened were also excluded from the study.

### 2.1. Study Design

All of the subjects were given an explanation of the study and then signed a written informed consent form, which was approved by the Institutional Review Board, before the treatments began. Drugs that had been used within the past month and those that were being used at the time were documented. During the study, the concomitant use of nonsteroidal anti-inflammatory drugs (NSAIDs), selective cyclooxygenase-2 (COX-2) inhibitors, acetaminophen, aspirin, and tricyclic antidepressants that were being taken before the study commenced was allowed, and their dosages were maintained during the study period. On the other hand, the initiation of any new analgesics other than the drugs under investigation was not allowed. However, the prophylactic use of antiemetics was allowed at the beginning of the treatment to prevent the nausea and vomiting that might be caused by opioid analgesics. Medications were allowed for the treatment of adverse events (AEs) even during treatment with the target drugs.

The subjects received the study drug (fentanyl matrix) or the comparison drug (gabapentin) after the first baseline assessment. The minimal dose (12 mcg/h) of the study drug was administered for six days, after which the pain was assessed every six days until day 28, during which time the dose was increased gradually by 12 mcg/h when the mean NRS was higher than 2. Finally, the last dose in the previous stage was maintained from days 29 to 56 (Table 1).

Gabapentin was administered at a dose of 300 mg once in the evening on day 1, 300 mg twice on day 2, 300 mg three times on days 3 and 4, and 300 mg both in the morning and afternoon and 600 mg in the evening on days 5 and 6. Subsequently, the dose was increased gradually by 300 mg per day up to 2400 mg/day when the mean NRS was higher than 2 during the pain assessments conducted every six days until day 28. Finally, the last dose in the previous stage was maintained from days 29 to 56. In the presence of possible AEs, the investigator was allowed to control the rate at which the dosage was increased (Table 1).

### 2.2. Evaluations and Statistical Analysis

The primary efficacy parameter of this study was the pain intensity difference (PID) from baseline at eight weeks after the first dose of the study drug (i.e., PID = NRS at baseline–NRS at day 57), with an objective to demonstrate that the study drug group was noninferior to the comparator group. Pain intensity was measured on an 11-point numeric rating scale (0 = no pain and 10 = the highest degree of pain). Secondary efficacy parameters that were also assessed were pain relief (a 6-point scale in which 4 = complete, 3 = fair, 2 = moderate, 1 = slight pain relief, 0 = no change, and -1 = pain worsening), Korean-Oswestry Disability Index (K-ODI), and Korean-Beck Depression Inventory (K-BDI). The ODI is widely used to estimate functional disability due to low back pain. It contains ten categories: intensity of pain, lifting, ability to care for oneself, ability to walk, ability to sit, sexual function, ability to stand, social life, sleep quality, and ability to travel. The BDI is a 21-question multiple-choice self-report inventory, one of the most widely used psychometric tests for measuring the severity of depression. The subjects visited the investigational site on days 29 and 57 for an assessment of any AEs caused by the drugs under investigation and the assessment of the efficacy parameters.

To decide the target sample size, variations due to changes in pain intensity in the study drug and comparator groups were calculated based on previous research, and the noninferiority margin (i.e., the minimal clinically significant difference) between the study drug and comparator groups in relation to the PID was estimated. The target was set at 58 subjects per group (116 subjects overall) based on the following assumptions: (1) a one-sided 2.5% (two-sided 5%) significance level, (2) a test power of 90%, (3) a minimal clinically significant difference (non-inferiority margin) in the PID of 1.39 between the study drug and comparator groups, (4) a change in the PID after eight weeks of 1.87 in both groups, (5) no difference in the PID between the groups in the population, (6) a follow-up loss of 30% in both groups during the study, and (7) 40 subjects per group for per-protocol (PP) analysis [24]. We conducted a *post hoc* power analysis to calculate the actual power of the study using the collected data and found that it was greater than 90% (nQuery Advisor ver. 6.01; Statistical Solutions Ltd., Cork, Ireland).

Subjects were considered to have completed the clinical trial when they had completed the third assessment on day 57. Subjects were removed from the study for the following reasons: (1) treatment was no longer possible due to AEs, (2) the subject decided to discontinue the treatment for safety reasons, (3) a major protocol violation occurred, (4) the subject did not cooperate with the procedures of the study, (5) the subject or his/her caregiver withdrew the consent to participate in the study, (6) the patient did not attend a follow-up, or (7) a female subject became pregnant.

The study subjects for whom the efficacy parameters were assessed were divided into intention-to-treat (ITT) and PP populations. The ITT population consisted of any subjects who met the inclusion/exclusion criteria at screening and who were randomized at baseline. The PP population consisted of subjects in the ITT population whose pain intensity was assessed at the third assessment after completing the study without removal for any of the reasons stated above.

The PID, primary efficacy parameter, was analyzed mainly for the PP population using one-sided 95% lower confidence limits for the difference in the mean PID, as measured between the baseline scores and the assessment scores on day 57, between the study drug and comparator groups. If the lower confidence limit was equal to or greater than the noninferiority margin of −1.39, pain reduction in the study drug group was judged to be noninferior to that of the comparator group. Similarly, if the one-sided 95% lower confidence limit exceeded 0, pain reduction in the study drug group was judged to be superior to that of the comparator group. The secondary parameters were analyzed mainly for the ITT population using the Mann–Whitney *U* test or *t*-test for continuous variables and Pearson's chi-square test or Fisher's exact test for dichotomous variables. The descriptive statistics for continuous variables were presented as means with standard deviation (SD), and dichotomous variables were presented as frequencies with percentages in parentheses.

For missing data, no imputations were performed, and the data were treated as missing for all variables. All statistical tests except for the noninferiority test were two-sided. *P* values lower than 0.05 were considered statistically significant. All analyses were conducted using the statistical software package SAS 9.X (Statistical Analysis System, SAS-Institute, Cary, NC, USA).

## 3. Results

### 3.1. Patients

**Figure 1:**
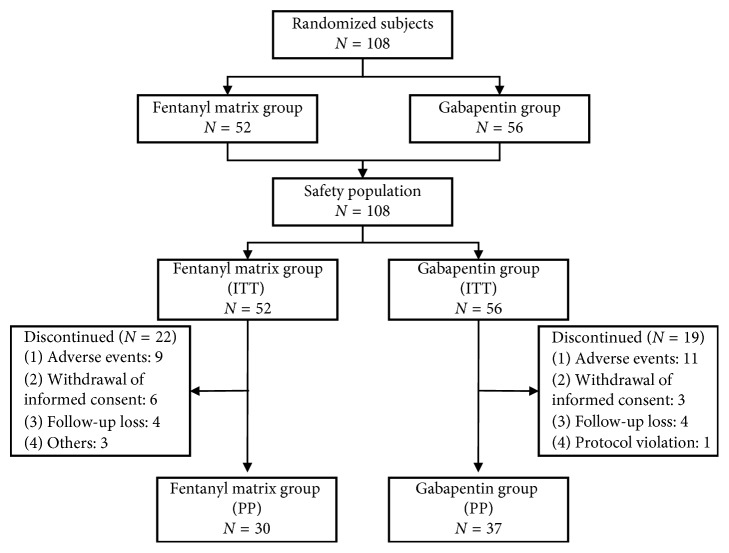
Categorization of the patients who participated in the study. ITT, intention-to-treat; PP, per protocol.

**Figure 2:**
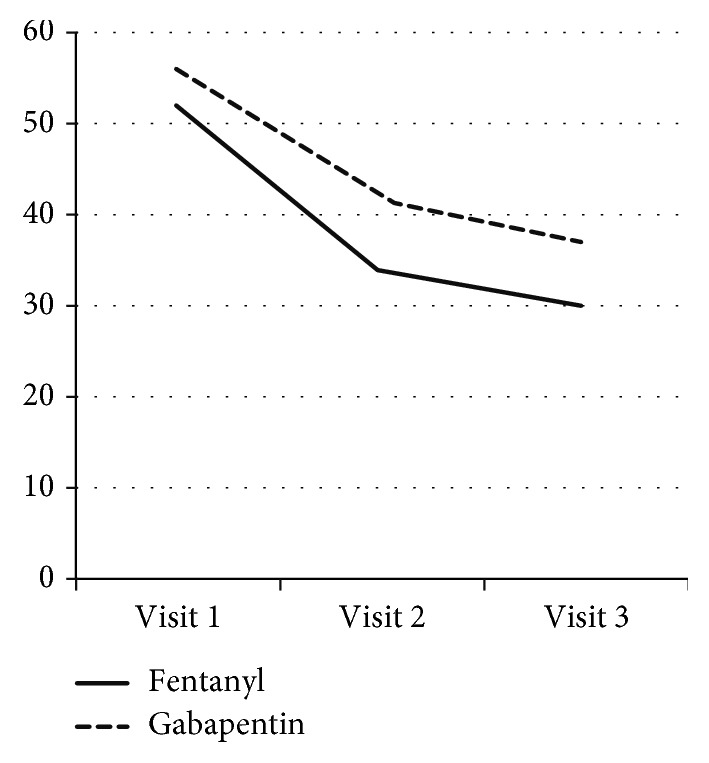
The number of patients remaining in the study over time.

Of the 108 subjects who provided informed consent and participated in this study, 67 (62.0%) completed the third assessment after receiving the study drug or comparator for 56 days and 41 (38.0%) were removed from the study (Figure 1). The most common reason for removal was AEs (20 subjects; 18.5%), followed by the withdrawal of informed consent (9; 8.3%), follow-up loss (8; 7.4%), others reasons (3; 2.8%), and a protocol violation (1; 0.9%). Thirty-two patients dropped out before visit 2, and 9 patients dropped out between visit 2 and the final visit (Figure 2).

Baseline age, weight, height, and mean pain intensity were not statistically significantly different between the two groups (Table 2). Spinal stenosis was the most commonly reported diagnosis for the cause of neuropathic pain in both groups. Concomitant analgesics other than the study drugs were used by 53.0% and 47.0% of patients in the study drug and comparator groups, respectively. The mean starting and final doses were 12.0 (±1.7) and 25.0 (±17.7) mcg/h, respectively, in the study drug group and 316.1 (±89.0) and 1580 (±583.3) mg, respectively, in the comparator group.

### 3.2. Primary Efficacy Analysis

**Figure 3:**
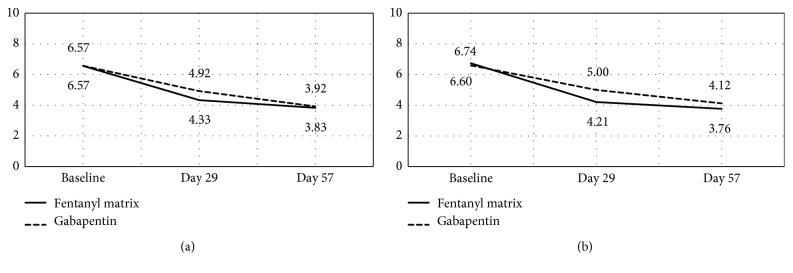
Change in pain intensity during the study period; ^a^*P* < 0.0001 (baseline vs. day 29); ^b^*P* < 0.0001 (baseline vs. day 57); ^c^*P*=0.0009 (baseline vs. day 29); ^d^*P* < 0.0001 (baseline vs. day 57). ^1^*P* < 0.0001 (baseline vs. day 29); ^2^*P* < 0.0001 (baseline vs. day 57); ^3^*P*=0.0006 (baseline vs. day 29); ^4^*P* < 0.0001 (baseline vs. day 57). (a) PP group. (b) ITT group.

In the PP population, the mean change in pain intensity from baseline at the final assessment, as assessed using the NRS, for the study drug and comparator groups, was 2.7 (±2.3) and 2.6 (±2.0), respectively. Because the lower confidence limit was −0.7935, which was higher than the noninferiority margin of −1.39, the decrease in mean pain intensity in the study drug group was judged to be noninferior to that in the comparator group at a one-sided 95% significance level (Table 3). Following this confirmation of noninferiority, a superiority analysis was performed with the PP population, but the decrease in pain intensity in the study drug group was not superior to that of the comparator group (*P*=0.8727). In the ITT population, the study drug and comparator groups demonstrated statistically significant decreases from baseline in the intensity of their pain at both the second and third assessments (Figure 3).

### 3.3. Secondary Efficacy Analysis

**Figure 4:**
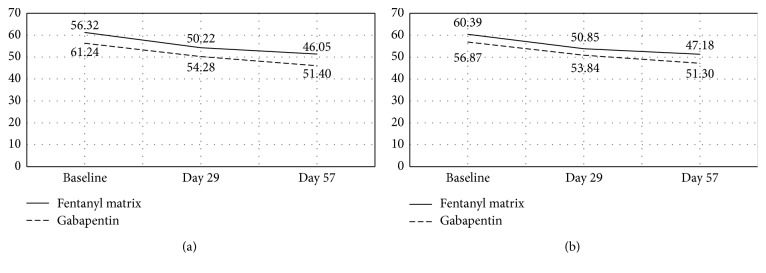
Change in the K-ODI during the study period; ^a^*P*=0.035090 (baseline vs. day 29); ^b^*P*=0.008163 (baseline vs. day 57); ^c^*P*=0.06822 (baseline vs. day 29); ^d^*P*=0.00340 (baseline vs. day 57). ^1^*P*=0.02996 (baseline vs. day 29); ^2^*P*=0.006848 (baseline vs. day 57); ^3^*P*=0.04832 (baseline vs. day 29); ^4^*P*=0.002508 (baseline vs. day 57). (a) PP group. (b) ITT group.

In the ITT population, there was no between-group difference in pain relief at the second and third assessments after treatment with the study drug and comparator (*P*=0.3359, Table 4). In the ITT population, the mean K-ODI score, which was used to compare the patients' functional status after treatment, was significantly lower than baseline at the second and third assessments in both groups (Figure 4), but there was no difference in the size of this reduction between the groups (*P*=0.8698, Table 5). Analysis of the change in the K-BDI score, which was used to evaluate depression levels among the patients, revealed no difference in the size of the decrease in K-BDI scores after treatment between the study drug and comparator groups (*P*=0.4095, Student's *t*-test).

### 3.4. Safety Analysis

In the safety analysis of the108 subjects who received the study drug or comparator at least once, 74 (68.5%) complained of AEs at least once, with 35 (67.3%) and 39 (69.6%) in the study drug and comparator groups, respectively. The most common AE was dizziness (41 subjects; 38.0%), while AEs that occurred in more than 10% of subjects included somnolence (34; 31.5%), nausea (23; 21.3%), constipation (18; 16.7%), vomiting (13; 12.0%), and asthenia (12; 11.1%; Table 6).

In the fentanyl group, nausea was the most common AE (19 subjects; 36.5%), followed by dizziness (16; 30.8%), somnolence (14; 26.9%), vomiting (11; 21.2%), and constipation (8; 15.4%). In the gabapentin group, dizziness (25 subjects; 44.6%) was the most common AE, while somnolence (20; 35.7%), constipation (10; 17.9%), and asthenia (7; 12.5%) occurred in more than 10% of subjects.

Twenty patients (18.5%) were removed from the study due to AEs (Figure 1). In both the ITT and PP populations, there was no statistically significant difference in the AE incidence rates between the fentanyl and gabapentin treatment groups (*P*=0.7049  and  0.7341, respectively).

## 4. Discussion

Antidepressants and anticonvulsants are conventional treatments for neuropathic pain. The currently recommended first-line treatments are anticonvulsants such as gabapentin and pregabalin, serotonin-norepinephrine reuptake inhibitors such as duloxetine and venlafaxine, and tricyclic antidepressants [25]. Although opioids are considered a second- or third-line treatment for neuropathic pain, controversy remains over their use [5–8]. In the past, opioids were perceived to be effective in controlling nociceptive pain but to have a limited effect on neuropathic pain [5–7]. In addition, they were not widely used because AEs often occurred before a concentration at which the pain was controlled could be reached [9–11]. However, recent studies have reported that opioids have a similar level of analgesic effect on neuropathic pain as they do on nociceptive pain [12–14]. In a systematic review of the efficacy of opioids in the treatment of neuropathic pain published by Eisenberg et al. in 2005 [15], although the evidence was not apparent in short-term use studies, opioids were significantly effective for neuropathic pain compared to placebo in intermediate-term use studies, and AEs were common but not life-threatening. The Cochrane review in 2013 [18] also reached a similar conclusion, but the analgesic efficacy of opioids for chronic neuropathic pain remained unclear because of the considerable variability in the type of neuropathic pain, type of opioids used, and duration of treatment in published RCTs.

The transdermal fentanyl delivery system is a formulation that is absorbed through the skin over an extended period. Transdermal fentanyl has been reported to be effective in relieving neuropathic and cancer pain, to have fewer constipation and sedative effects, and to lead to significantly higher patient satisfaction because of the convenience of application. Fentanyl matrix patches have also been found to relieve neuropathic pain that is not sensitive to other opioids and has been reported to be effective and safe for opioid-naive patients [18–21].

We believe that fentanyl matrix may be a good alternative to oral drugs for neuropathic pain in primary clinical settings. Although several reports have compared gabapentin to oral opioids, few comparisons have been made between anticonvulsants and transdermal fentanyl in terms of efficacy and safety. Therefore, we decided to compare fentanyl matrix with gabapentin even though these agents are administered via different routes and have different pharmacokinetic profiles. To our knowledge, this is the first RCT comparing transdermal fentanyl with gabapentin in patients with chronic noncancer neuropathic pain.

The efficacy of gabapentin in relieving neuropathic pain has been proven in many studies and is recommended as a first-line treatment [22, 23, 25]. Because of this, our study was designed as a noninferiority trial. In terms of the change in pain intensity, which was the primary endpoint of this study, transdermal fentanyl was found to be noninferior to gabapentin. In addition, the secondary endpoints—K-ODI score, K-BDI score, and safety—were not statistically different between the two groups. Although transdermal fentanyl was not superior in the treatment of neuropathic pain of radicular origin, there was no significant difference between the treatments, particularly in terms of AEs, which indicates that fentanyl patches could be used as a first-line treatment for specific populations.

The number of dropouts due to AEs was lower in the fentanyl group (9 subjects compared to 11 in the gabapentin group). The most serious issue that arises during treatment with strong opioids is that patients often voluntarily discontinue treatment due to AEs such as nausea, vomiting, and constipation. The significance of transdermal fentanyl in our study is that the incidence of early AEs is not different from that existing in first-line treatments. With the recent focus on the misuse and abuse of prescription drugs, there is a trend for strong opioids (e.g., oxycodone, morphine, fentanyl, hydromorphone, buprenorphine, and methadone) to be classified as third-line treatments, which warrants long-term follow-up studies [8].

Although pain intensity frequently is measured on an 11-point pain intensity NRS, it is difficult to interpret the clinical importance of minor changes from baseline using this scale (e.g., 1- or 2-point changes). Farrar et al. [26] reported that, on average, a reduction of approximately two points or of approximately 30% in the NRS score represented a clinically important difference. They also found that the relationship between the percentage change and patient global change of impression was also consistent regardless of the baseline pain, while higher baseline scores required larger raw changes in order for a clinically important difference to be recognized. They recommended that applying these results in future studies may provide a standard definition for clinical improvement in clinical trials of chronic pain therapies. Therefore, we chose to evaluate the PID to improve the comparability, validity, and clinical applicability of the present study. In addition, Fischer et al. [27] compared the relative responsiveness to treatment effects of the change in pain intensity scores between pretreatment and posttreatment (on a 100 mm visual analogue scale) and a seven-point retrospective rating of deterioration/improvement (rating from “very much worse” to “very much better”). They reported that the global retrospective ratings of improvement were more responsive to treatment effects than were the changes in pain intensity scores. We thus chose pain relief as a secondary efficacy parameter to evaluate any difference between the two drugs that may not have been detected by the primary efficacy parameter.

The dropout rate was relatively high in this study (38.0%). The dropout rate due to AEs was 18.5%, which was similar to that of previously reported studies [28, 29]. A substantial number of patients withdrew their informed consent (8.3%) and did not attend follow-up without a specific reason (7.4%). We cannot explain exactly what the cause is for the latter patients. However, we believe that the dropout rate of our study was not so high compared to those of other randomized trials [28, 29]. We confirmed that there were no marked differences in the reasons for dropping out of the study between the two treatment (Figure 1), indicating that the results would be relatively consistent between the ITT and PP populations. In our study protocol, we based the final study conclusions on the primary efficacy analysis using the PP population.

Our results should be interpreted carefully based on the study design. For example, the raters of the questionnaires used in this study were blinded to the treatments, but the subjects were not. A double-dummy or double-blind design might have overcome this bias, but a comparison of the two medications with completely different routes of administration without using a placebo has its inherent limitations. In addition, patients who had received an extended-release opioid within the past 30 days were excluded from the study, but the remaining patients were not necessarily all opioid-naive. Strong opioids generally are recommended for patients who do not respond to treatments with conventional NSAIDs or weak opioids. If the target patients were narrowed down to opioid-native patients or those who did not respond to conventional opioids, the incidence of AEs would be higher or the effect of study drugs would decrease, respectively. In addition, differences between routes of administration, mechanism of action, alterations in absorption/drug levels, and variability between formulations (brand vs. generic) also are limitations of the current study.

We did not exclude subjects who were on antidepressants, which may have had a role in the treatment of neuropathic pain, because they are widely used for purposes other than pain relief. Other analgesic drugs that the patients were allowed to take may have interacted differently with the drugs under investigation and significantly influenced the outcomes. In addition, the frequency of intake, the clearance time, and other pharmacokinetic parameters related to these drugs might have affected the findings. However, patients were prohibited from starting new drug treatments during the study period.

The presence of radiculopathy was confirmed using EMG for all of the patients, and they were all judged to be experiencing neuropathic pain based on S-LANSS assessment. However, it is difficult to apply these criteria when prescribing medications in real-world clinical settings. A number of patients with a spinal degenerative disease experience both nociceptive and neuropathic pain [30], which should be considered in the interpretation and generalization of our results. Because opioids are known to affect both nociceptive and neuropathic pain, they may be preferred when a patient has nociceptive-dominant pain.

The outcomes observed from the administration of the two different drugs mostly overlapped in this study. Opioid and anticonvulsant have different mechanisms in relieving neuropathic pain. We believe that there was no significant difference in the clinical results between the two groups because we focused on the neuropathic features of lumbosacral radiculopathy. If the target pain had included a nociceptive component such as low back pain, the outcomes might have been different. In addition, although the overall incidence of AEs did not differ between the two groups, dizziness and somnolence were more frequent in the gabapentin group, whereas nausea and vomiting were more frequent in the fentanyl patch group.

Despite the increasing prescription of fentanyl for chronic, cancer, and postoperative pain, little research has been conducted on its effect on neuropathic pain. A Cochrane review in 2016 [18], in which only one clinical trial met the inclusion criteria, concluded that there was not enough evidence for the effectiveness of fentanyl in controlling neuropathic pain. In a study comparing the effectiveness of pregabalin and transdermal fentanyl in patients with neuropathic cancer pain, Raptis et al. [31] reported that significantly more patients in the pregabalin group had a reduction in pain of more than 30%. In research evaluating the efficacy and safety of fentanyl transdermal patches, Park et al. [19] argued that they were effective for moderate-to-severe chronic noncancer pain including neuropathic pain but failed to present clear criteria for neuropathic pain.

Although lumbar radiculopathy is a common disease with neuropathic features, few clinical trials have been conducted for various medications targeting neuropathic pain of radicular origin. Given that transdermal fentanyl was noninferior to gabapentin, which is a first-line treatment for neuropathic pain, our research indicates that transdermal fentanyl could be beneficial in terms of efficacy and safety for patients who are not comfortable with oral gabapentin. However, further studies are warranted to determine the appropriate treatment guidelines.

## 5. Conclusions

This clinical trial comparing fentanyl matrix and gabapentin in patients with chronic neuropathic pain demonstrated that both drugs were equally effective in reducing neuropathic pain. The pain reduction in the fentanyl matrix group was noninferior to that of the gabapentin group, with no difference between the two groups in terms of patient functional status, depressive symptoms, and the occurrence of adverse events.

## Figures and Tables

**Table 1:** Drug administration schedule for the present study.

Day	1–6				7–28	29–56
Fentanyl	12 mcg/h				Pain was assessed every six days. The dose was increased gradually by 12 mcg/h when the mean NRS was higher than 2.	The last dose was maintained
*Day*	*1*	*2*	*3–4*	*5–6*	*7–28*	*29–56*
Gabapentin	300 mg hs	300 mg bid	300 mg tid	Morning: 300 mg, afternoon: 300 mg, evening: 600 mg	Pain was assessed every six days. The dose was increased gradually by 300 mg/day up to 2400 mg/day when the mean NRS was higher than 2.	The last dose was maintained

**Table 2:** Baseline demographics and clinical characteristics.

Characteristics	Fentanyl matrix (*n* = 52)	Gabapentin (*n* = 56)	Total (*n* = 108)
Mean (SD) age^1^ (y)	59.0 (13.1)	58.2 (12.1)	58.6 (12.6)
Gender, *n* (%)			
Female	30 (57.7)	30 (53.6)	60 (55.6)
Male	22 (42.3)	26 (46.4)	48 (44.4)
Mean (SD) weight^2^ (kg)	63.1 (9.7)	65.5 (15.8)	64.3 (13.2)
Mean (SD) height^3^ (cm)	161.4 (9.0)	162.1 (7.9)	161.8 (8.4)
Mean (SD) pain intensity^4^ (NRS)	6.8 (1.8)	6.5 (1.8)	6.6 (1.8)

^1^ *P*=0.7255; ^2^*P*=0.3293; ^3^*P*=0.6796; ^4^*P*=0.4968; SD: standard deviation; NRS: numeric rating scale.

**Table 3:** Pain intensity difference between the groups.

Analysis population	*N*	Mean (SD)	Mean difference (one-sided 95% CI)	*P* value^1^
ITT				
Fentanyl matrix	34	3.0 (2.3)	0.5 (−0.33, ∞)	0.5660
Gabapentin	42	2.5 (2.0)		

PP				
Fentanyl matrix	30	2.7 (2.3)	0.1 (−0.79, ∞)	0.4145
Gabapentin	37	2.6 (2.0)		

^1^Student's *t*-test; SD: standard deviation; CI: confidence interval.

**Table 4:** Pain relief between groups.

Analysis population	*N*	Mean (SD)	*P* value^1^
ITT			
Fentanyl matrix	34	1.21 (1.17)	0.3359
Gabapentin	42	0.95 (1.08)	

PP			
Fentanyl matrix	30	1.07 (1.17)	0.8131
Gabapentin	37	1.00 (1.11)	

^1^Student's *t*-test; SD: standard deviation.

**Table 5:** Difference in the mean K-ODI scores between treatments.

Analysis population	*N* evaluable	Mean (SD)	Mean difference (one-sided 95% CI)	*P* value^1^
ITT				
Fentanyl matrix	34	9.1 (18.2)	−0.6 (−7.91, 6.71)	0.8698
Gabapentin	42	9.7 (12.3)		

^1^Student's *t*-test; K-ODI: Korean–Oswestry Disability Index; SD: standard deviation; CI: confidence interval.

**Table 6:** Incidence of AEs in ≥5% of patients by treatment.

AE, *n* (%)	Fentanyl matrix (*N* = 52)	Gabapentin (*N* = 56)	Total (*N* = 108)
Dizziness	16 (30.8%)	25 (44.6%)	41 (38.0%)
Somnolence	15 (28.8%)	21 (37.5%)	36 (33.3%)
Nausea	19 (36.5%)	4 (7.1%)	23 (21.3%)
Constipation	8 (15.4%)	10 (17.9%)	18 (16.7%)
Vomiting	11 (21.2%)	2 (3.6%)	13 (12.0%)
Asthenia	7 (13.5%)	8 (14.3%)	15 (13.9%)
Headache	3 (5.8%)	4 (7.1%)	7 (6.5%)

AE: adverse event.

## Data Availability

The data used to support the findings of this study are included within the article.
